# Salinity tolerance in the halophyte species *Cakile maritima* from the Apulia region, southern Italy

**DOI:** 10.3389/fpls.2025.1662491

**Published:** 2025-09-01

**Authors:** Giulia Conversa, Lucia Botticella, Corrado Lazzizera, Anna Bonasia, Luigi Giuseppe Duri, Antonio Elia

**Affiliations:** Department of Agriculture, Food, Natural Resources and Engineering (DAFNE), University of Foggia, Foggia, Italy

**Keywords:** sea rocket, leaf gas exchanges, chlorophyll fluorescence, cations, chlorophylls, phenols, ascorbic acid, antioxidant capacity

## Abstract

**Introduction:**

*Cakile maritima* is a succulent halophyte from the Brassicaceae family, commonly found along sandy coasts. Understanding its response mechanisms to sodium excess is crucial for its exploitation under sustainable biosaline farming.

**Methods:**

For the first time, this research investigated the pinnatifid *C. maritima* population from the Apulia region (Italy) grown under varying levels of NaCl (0 -T0, 100 -T100 and 400 -T400 mM NaCl).

**Results:**

The T100 plants showed higher leaf area (LA) and specific leaf area (SLA) compared to T0, with a slight reduction in succulence index (SI). In T400 plants, a reduction in shoot and root fresh weight, water content (WC), leaf dry weight, LA, and SLA was observed, alongside an increase in SI and dry matter concentration. No changes were detected in leaf Na and Cl concentrations, whereas T400 stems accumulated Na. Leaf K, Mg, and Ca concentrations remained stable. The operating efficiency of PSII (ΦPSII) was similar across treatments. In salt-exposed plants, the decrease of Fv’/Fm’ was counteracted by an improvement of qP, with carotenoids and anthocyanins appearing to be involved in photoprotection. Salt-exposed plants maintained stomatal opening (gs), allowing a higher CO2 assimilation rate (A_n_), especially in T100. Despite unimpaired A_n_, T400 plants exhibited reduced canopy-level photosynthesis due to lower LA, leading to reduced shoot biomass. Among antioxidants, ascorbic acid and anthocyanins were effective in improving the antioxidative defence of T400 plants.

**Discussion:**

The results indicate that *C. maritima* employs a complex protective strategy involving morphological adjustments, selective ion accumulation, efficient photoprotection, maintained gas exchange, and a potent antioxidant system to mitigate salinity stress, demonstrating its strong potential for biosaline agriculture.

## 1 Introduction

Globally, nearly 20% of cultivated land faces soil salinization, which is expected to increase to 50% by 2050 (Mann et al., 2023; Bazihizina et al., 2024). The impact of salinity stress on plant growth and development is substantial, affecting various aspects, including seed germination, plant anatomy and physiology, root structure, protein synthesis, and gene expression. This effect varies based on the level of salinity, the duration of exposure, and the specific plant species. With short-term exposure to salinity, the elongation rates of plant cells decrease. However, prolonged exposure to salinity reduces cell division, restricting plant growth and causing visible injuries, such as leaf death. High Na and Cl levels in soil limit the uptake of essential macronutrients, such as K, Ca, and Mg, thereby disrupting physiological functions. Salinity also negatively affects photosynthesis by damaging pigments, lowering stomatal density and conductance, and increasing mesophyll resistance, thus hampering the efficiency of photosystems I and II (Rahman et al., 2021). Salt stress also decreases the activity of RuBisCO, a crucial enzyme for CO_2_ assimilation, and leads to the production of reactive oxygen species (ROS) in cellular organelles, causing protein synthesis reduction, cell membrane damage, and instability in the photosynthetic apparatus (Rahman et al., 2021).

Halophytes represent a small group (fewer than 1% of all plant species) that exhibit a natural ability to resist or tolerate salinity levels ranging from 50 to 500 mM NaCl. These plants may require consistent saline conditions for optimal growth (obligate halophytes) or can endure certain levels of salinity (facultative halophytes), although they typically thrive better in non-saline or low-salinity environments (Rahman et al., 2021; Bazihizina et al., 2024).

The remarkable ability of halophytes to flourish in saline soils is mainly achieved through physiological mechanisms such as salt exclusion at the root level, salt excretion via specialized epidermal structures (e.g., salt glands and bladder cells), and salt inclusion. However, one or more of these features can be present in different halophytes to manage salinity (Rahman et al., 2021). The salt inclusion strategies aim to dilute absorbed salts within succulent leaves and stems using inherent mechanisms (ion compartmentalization, osmotic adjustments, antioxidative defense systems, redox homeostasis) (Mann et al., 2023).

*Cakile marittima* is an annual succulent halophyte belonging to the Brassicaceae family, abundantly found along the sandy coasts of the Atlantic and Mediterranean regions. Plants can potentially be used for food and industrial (high-healthy foods, pharmaceutical, oilseed extraction) purposes (Arbelet-Bonnin et al., 2019; Conversa et al., 2024), offering a valuable tool for sustainable biosaline farming or the exploitation of saline environments (Arbelet-Bonnin et al., 2019; Bazihizina et al., 2024). Additionally, it could be used as a plant model for salinity tolerance studies (Ben Hamed et al., 2016; Mir et al., 2024). Deciphering the response mechanisms in halophytes under conditions of sodium excess-induced tolerance and/or toxicity holds significant value for optimizing agricultural practices in saline environments, thereby maximizing both yield and the production of health-promoting secondary metabolites.

To the best of our knowledge, research on the salinity tolerance strategies of *C. maritima* has been conducted substantially on ecotypes of Tunisia. It highlighted that this species is a salt-including euhalophyte as its growth is improved by moderate soil salinity (50–100 mM NaCl) and it exhibits no adverse effects even at salinity levels as high as 200 mM NaCl (Debez et al., 2004, 2006, 2008, 2012; Arbelet-Bonnin et al., 2019; Ben Amor et al., 2020). In proportion to the external concentration, it can take up a large amount of Na and, to a lesser extent, of Cl. Sodium can be efficiently removed from the cytosol mainly through compartmentalization in vacuoles, since no epidermal salt excretion was observed (Debez et al., 2004, 2006). Na leaf accumulation has been positively correlated to the improved plant water status, leaf thickness and succulence (Debez et al., 2004, 2006; Ben Hamed et al., 2016). Moreover, despite a reduction of K, Ca, and Mg uptake (Debez et al., 2004, 2008) or no changes in K accumulation (Ben Hamed-Louati et al., 2016) have been observed, a selective K uptake has been suggested to regulate Na/K ratio. Photosynthesis (Debez et al., 2006) and chlorophyll fluorescence (Debez et al., 2008, 2012; Arbelet-Bonnin et al., 2020) parameters pointed out that carbon assimilation rate and PSII efficiency were improved under moderate NaCl concentration, while decreasing when plants experienced higher salinity. Similarly, both the enzymatic and non-enzymatic antioxidant systems were elicited depending on salinity pressure for leaf ROS detoxifying (Ben Amor et al., 2006, 2007; Ksouri et al., 2007; Ellouzi et al., 2011, 2014; Mansour et al., 2018). Among non-enzymatic antioxidants, ascorbate (Ben Amor et al., 2006, 2007; Ellouzi et al., 2014; Ben Hamed-Louati et al., 2016), phenols (Ksouri et al., 2007; Mansour et al., 2018) and carotenoids (Mansour et al., 2018) have a pivotal role in contrasting oxidative stress. The occurrence of these responses and their efficiency have been related to specific bioclimatic conditions of *C. maritima* ecotypes, with the arid-adapted populations being more tolerant to salinity exposure than the plants adapted to a humid habitat (Ben Amor et al., 2006; Ksouri et al., 2007; Megdiche et al., 2007).

In Italy, *C. maritima* subsp. *maritima* is spread along sandy coasts, with some studies exploring physiological (Gratani et al., 2009), morpho-functional (Ciccarelli et al., 2010) and biochemical (Conversa et al., 2024) adaptation in natural areas. Both in Tuscany (Ciccarelli et al., 2010) and Puglia (Conversa et al., 2024) regions, the diffusion of two morphotypes distinguished by entire or pinnatifid leaves has been ascertained (Ciccarelli et al., 2010) with the pinnatifid type appearing to be more adapted to the coastal climate of southern Italy (Conversa et al., 2024). Nevertheless, to date, no studies have been conducted specifically on their salinity tolerance/resistance. To gain knowledge on the salt response mechanisms of this species, the present work aims to explore the morpho-biophysiological traits, mineral nutrition and antioxidative characteristics of the pinnatifid population of *C. maritima* from the Apulia region grown at increasing NaCl levels.

## 2 Materials and methods

### 2.1 Experimental location and set-up

The experiment was conducted in an unheated glass greenhouse (model Tulip 6, Serra e Giardini Mirandola, MO, Italy) at the Department of Agriculture, Food, Natural Resources, and Engineering (DAFNE) (University of Foggia), located in Foggia, Puglia, southern Italy (latitude 41° 46′ N, longitude 15° 55′ E, 74 m above sea level). The experiment took place during February and March 2024. In the greenhouse, the mean minimum and maximum daily air temperature ranged from 10-11°C and 20-22°C, respectively. The relative humidity varied between 70 and 80%. Seeds of *C. maritima* were collected in September 2023 from Margherita di Savoia (BT), northern Puglia (latitude, 41.4° N; longitude, 16.0° E; altitude, 0–5 m a.s.l.). The area has a Mediterranean climate characterized by mild winters and hot, dry summers. Over the past thirty years, the average recorded temperatures have been a minimum of 9.8 ± 1.6°C and a maximum of 21.6 ± 2.1°C. The mean temperatures for the coldest and hottest months are 7.5°C in January and 25.0°C in July, respectively. Annual rainfall averages 497 mm, with 33% occurring from October to December, 25% from January to March, 22% in spring, and only 11% during July and August.

Seeds were sown in pots (0.72 L) filled with a standardized growth medium consisting of peat (Brill 3 TYPical, Gebr. Brill Substrate GmbH & Co. KG, Georgsdorf, NI, Germany), sieved sandy soil(collected from the same location as the seeds), and perlite (Agrilit3, Perlite Italiana s.r.l., Corsico, Milano, Italy). The physicochemical analysis of the sandy soil is reported in the **Supplementary Table 1**. The ratio of these components was 50:30:20. The pots were irrigated with tap water. Two-week-old seedlings, one per pot, were watered twice a week using a diluted (two-fold) Hoagland nutrient solution (NS). The chemical characteristics of the tap water used for irrigation and for NS production are reported in the **Supplementary Table 2**.

After a one-month pretreatment phase, plants with an average weight of 1.58 ± 0.48 g (height 8.4 ± 1.2 cm) were divided into three groups. The first group, which served as the control (T0), received a full-strength NS without sodium chloride (NaCl). The second group was irrigated with the same NS containing 100 mM NaCl, while the third group received the NS with 400 mM NaCl. To prevent osmotic shock, plants were top-irrigated for three weeks with NS at salt levels that were increased by 50 mM NaCl, until the desired salt concentration was achieved. This ensured a gradual transition to higher salinity. In total, each plant received 350 mL of T0, T100 and T400 NS. Treatments were arranged using a randomized block design with three replicates. The experimental unit consisted of 12 pots each containing one plant. Therefore, in total 36 plants (12 plants x 3 replications) were grown for each treatment. The plants were harvested three days after the last treatment application.

At the end of the trial, the electrical conductivity of the substrate was measured at 3.6 (± 0.07), 4.0 (± 0.03) and 6.9 (± 0.16) dS m^-1^, for treatments T0, T100 and T400, respectively.

### 2.2 Plant material, bio-physiological and chemical determinations

On 9 plants belonging to each experimental unit, the aerial biomass was distinguished in leaves, tender and coriaceous stems and roots. Number, area and fresh weight of leaves of each plant were determined. Leaf dry weight (DW) was determined by drying in a thermoventilated oven at 70°C until a constant mass was achieved. Fresh weight (FW) and dry weight (DW) for the stem and root were also measured. The dry matter (DM) content of leaves, stems, and roots was calculated as (DW/FW) × 100. Leaf area was measured by scanning leaves at 360 dpi using an Epson Perfection V750 PRO scanner (Epson Italia S.p.A., Cinisello Balsamo, MI, Italy). The scanned images were processed with the ImageJ software (National Institutes of Health, Bethesda, MD, USA). Specific leaf area (SLA) was determined as the ratio of leaf area to dry weight (LA/DW), while leaf mass per unit leaf area (LMA) was calculated as DW/LA. Furthermore, the succulence index (SI) was computed as the ratio of the difference between fresh and dry weight to leaf area, following Gratani et al. (2009). The water content (WC) of leaves, stems, and roots was determined as the ratio of the difference between fresh and dry weight to fresh weight. Six subsamples of leaves and stems per experimental unit were lyophilized (ScanVac CoolSafe 55–9 Pro; LaboGene ApS, Lynge, Denmark) for subsequent phytochemical analyses.

#### 2.2.1 Chlorophyll fluorescence and leaf gas exchange

Leaf gas exchange and chlorophyll *a* fluorescence parameters were assessed on three plants per experimental unit. For each plant, two fully expanded leaves were selected for evaluation. Gas exchange parameters included net photosynthetic rate (A_n_), intercellular CO_2_ concentration (C_i_), stomatal conductance (g_s_), and transpiration rate (E). Chlorophyll fluorescence parameters included the effective quantum yield of photosystem II (ΦPSII), photochemical quenching (qP), and the maximum efficiency of PSII photochemistry in the light (Fv′/Fm′). All measurements were performed using an open gas exchange system equipped with a leaf chamber fluorometer and LED light source (LI-6400XT, LI-COR Inc., Lincoln, NE, USA). Preliminary light response curves were generated by exposing leaves to increasing photosynthetically active radiation (PAR) from 0 to 2,500 µmol m^-2^ s^-1^ (data not shown). Subsequent measurements were carried out under standardized saturating light conditions: PAR at 2,000 µmol m^-2^ s^-1^, leaf temperature maintained at 23 °C, ambient CO_2_ concentration set at 400 µmol mol^-1^, and an air flow rate of 300 cm³ s^-1^.

#### 2.2.2 Inorganic ions and ascorbic acid

For the extraction of inorganic cations, 0.3 g of lyophilized samples of leaves and stems, previously ashed in a muffle furnace at 550°C for 6 hours, were subjected to acid digestion using 20 mL of 1 mol L^-1^ HCl, followed by boiling for 30 minutes. The digested solution was then filtered through a 0.22 µm Millipore filter, diluted, and analyzed by ion chromatography. This analysis was performed using a self-regenerating DRS-600 suppressor (4 mm), a Dionex IonPack CS12A analytical column (4 mm × 250 mm, 5 µm), and an eluent consisting of 20 mM methane-sulfonic acid, with a flow rate of 1 mL min^-1^. Inorganic anions were extracted from 0.5 g of lyophilized samples of leaves using 50 mL of eluent solution, followed by agitation in a shaking water bath at room temperature for 30 minutes. The resulting mixture was filtered twice through Whatman No. 2 filter paper, then further filtered through a 0.22 µm Millipore filter. The filtered extract was then injected into an ion chromatography system (ICS-3000, Dionex Corp., Milan, Italy), which was equipped with an isocratic pump, AS-DV autosampler, a self-regenerating ASR anion suppressor (4 mm), and a Dionex Ion-Pac AS23 analytical column (4 mm × 250 mm) along with a guard column (4 mm × 50 mm) maintained at 35°C. The mobile phase was composed of 3.5 mM sodium carbonate and 1 mM sodium bicarbonate, with a flow rate of 1 mL min^-1^. Cation and anion compounds were identified by comparing their retention times with those of standard solutions. Peak areas were analyzed using Dionex Chromeleon software (version 6.80, ThermoFisher Scientific, Waltham, MA, USA) (Cataldi et al., 2003). Leaf ascorbic acid content was measured with a reflectometer (RQflex 10 plus, Merck, Germany) using a 25–450 mg L^−1^ measuring range for ascorbic acid. The method consists of reducing yellow molybdophosphoric acid to molybdenum blue by the action of ascorbic acid (Balık et al., 2023).

#### 2.2.3 Chlorophylls, carotenoids, and anthocyanins

Chlorophyll (Chl) (a, b, and total) and carotenoid concentrations were determined spectrophotometrically according to the method described by Sumanta et al. (2014), with slight modifications. Lyophilized leaf tissue (0.05 g) was extracted with 1.5 mL of 80% ethanol (ethanol:water, 80:20 v/v) containing 0.1% hydrochloric acid in 10 mL screw-cap tubes. Samples were subjected to ultrasonic extraction using an ultrasonic cleaner (DU-32 Digital; Argo Lab, Carpi, MO, Italy) at room temperature for 30 minutes. Following sonication, the samples were centrifuged at 4,000 × g for 15 minutes at 4 °C in a refrigerated centrifuge (Beckman Coulter Allegra™ 25, Fullerton, CA, USA). The supernatant was collected, and the extraction procedure was repeated twice. The combined supernatants were then filtered through 0.22 µm reinforced nylon membrane filters. The extract solution was then analyzed using a spectrophotometer (Evolution 201 UV-Visible Spectrophotometers, Thermo Scientific Waltham, MA, USA) at wavelengths of 664, 649 and 470 nm. The following equations were used for quantification: Chl a=13.36(A664)-5.19 (A649); Chl b=27.43(A649)-8.12(A664); Chl total=17.32(A649)+7.18(A664). Carotenoids were calculated using the equation: Carotenoids (1000(A470)-2.13Chl a-97.63Chl b)/209. Anthocyanin content was quantified using the same extract, following the method of Sims and Gamon (2002). Absorbance was measured at 525 nm and 650 nm, and chlorophyll interference was corrected using the equation: AA=A529-(0.288*A650), where AA represents the corrected anthocyanin absorbance. Total anthocyanin content was expressed as cyanidin-3-glucoside (c.g.) equivalents per unit weight, calculated using a molar extinction coefficient of 26,900 L mol^-1^ cm^-1^ at 525 nm (Murray and Hackett, 1991).

#### 2.2.4 Total phenols and flavonoids

Phenols extraction was performed on lyophilized leaf samples (0.3 g) using 1 mL of an 80% methanol solution at room temperature in an ultrasonic cleaner bath (DU-32 Digital; Argo Lab, Carpi, MO, Italy) for 15 minutes. The mixture was then centrifuged at 14,000 rpm for 15 minutes at 4°C in a refrigerated centrifuge (ThermoFisher Scientific, Waltham, MA, USA), and the supernatant was collected, while the residual pellet underwent a second extraction. The extracts were stored at -20°C and analyzed within 24 hours. Total polyphenols (TP) were determined by spectrophotometric analysis according to the method of Singleton and Rossi (1965): 0.1 mL of the extracts were diluted with 3 mL of distilled water, after which 0.5 mL of Folin-Ciocalteu reagent was added and the mixture was kept at room temperature for 5 minutes. Then, 1.0 mL of 20% Na2CO3 was added to the mixture. After 45 minutes at 30°C, the absorbance of the resulting solutions was measured at 750 nm using a spectrophotometer (Shimatzu UV-1800, Shimadzu Scientific Instruments, North America, USA). The values were determined from a calibration curve (0–250 mg L-1; R² 0.998) prepared with standard gallic acid (g.a.) solutions, applying the same procedure used for the extracts. The results were expressed as gallic acid equivalents (g.a.e.) per unit weight. Flavonoids extraction was performed on lyophilized samples (0.3 g) using 1 mL of an 80% methanol solution at room temperature in an ultrasonic cleaner bath (DU-32 Digital; Argo Lab, Carpi, MO, Italy) for 15 minutes. The mixture was then centrifuged at 14,000 rpm for 15 minutes at 4°C in a refrigerated centrifuge (ThermoFisher Scientific, Waltham, MA, USA), and the supernatant was collected, while the residual pellet underwent a second extraction. The extracts were stored at -20°C and analyzed within 24 hours. The flavonoid content was quantified using the AlCl3 colorimetric method (Chandra et al., 2014). Briefly, 200 µL of the extract or diluted quercetin standard solutions were mixed with 40 µL of 10% AlCl3, 40 µL of 1 M potassium acetate, and 1,120 µL of distilled water. The mixture was incubated at room temperature for 30 minutes, and absorbance was measured at 415 nm using a UV-Vis spectrophotometer (Shimadzu UV-1800, Shimadzu Scientific Instruments, North America, USA). The total flavonoid content was expressed as quercetin equivalents (q.e.) per unit weight.

#### 2.2.5 Antioxidant capacity

##### 2.2.5.1 Radical scavenging capacity by the TEAC (ABTS) assay

Hydrophilic and lipophilic components were extracted from finely ground lyophilized leaf samples. To 0.3 g of each sample, 1 mL of 80% methanol was added, and the mixture was agitated for 15 minutes. The supernatant, containing the hydrophilic fraction, was recovered after centrifugation at 13,000 rpm for 10 minutes, while the pellet was re-extracted in the same manner. For the lipophilic fraction extraction, 1 mL of hexane was added to the pellet. The suspension was placed in an ultrasonic bath and subsequently centrifuged as previously described. The recovered supernatants were then combined. Both fractions’ extracts were stored at -20°C until analysis. The antioxidant activity of both the hydrophilic and lipophilic fractions was assessed using the TEAC (Trolox Equivalent Antioxidant Capacity) assay, as described by Re et al. (1999). Specifically, the ability of the antioxidants to reduce the ABTS•+ radical cation was measured spectrophotometrically at a wavelength of 734 nm, indicated by a decrease in absorbance. The results are expressed as Trolox equivalent antioxidant capacity, derived from the use of the Trolox reagent (6-hydroxy-2,5,7,8-tetramethylchroman-2-carboxylic acid) to construct the calibration curve. The Trolox stock solution was 1,100 µM, and the concentration range of the calibration curve was 0-15 µM.

##### 2.2.5.2 Radical scavenging capacity by the DPPH assay

The DPPH assay (2,2-diphenyl-1-picrylhydrazyl) was applied exclusively to hydrophilic antioxidants (only for methanolic extracts), following a modified method described by Lisanti et al. (2015). This method is based on the use of the free radical DPPH. The unpaired electron in the DPPH radical produces a characteristic absorbance peak at 517 nm. The DPPH radical was progressively solubilized in ethanol to achieve a concentration that produced an absorbance between 1.12 and 1.08 at 517 nm. The analytical solution was obtained by mixing 1.9 mL of diluted DPPH and 0.1 mL of the sample extract or standard Trolox solution. The Trolox stock solution was 1,000 µM and the concentration range of the calibration curve was 0-800 µM. Absorbance was measured at 517 nm after 30 minutes, with the sample kept in the dark. The values were determined from a calibration curve prepared with Trolox solutions, following the same procedure used for the sample extract. The results were expressed as Trolox Equivalent (T.E.) per unit weight.

##### 2.2.5.3 Antioxidant activity assessed by the FRAP assay

The antioxidant activity was evaluated using a slightly modified version of the Ferric Reducing Antioxidant Power (FRAP) assay described by Lisanti et al. (2015), a method applied to both hydrophilic and lipophilic antioxidants. This assay is based on the ability of antioxidants to reduce Fe^3+^ ions to Fe^2+^ through an electron transfer mechanism in the presence of the TPTZ (2,4,6-tripyridyl-s-triazine) solution. The reaction creates a blue Fe^2+^–TPTZ complex that exhibits a maximum absorbance at 593 nm. This reaction is pH-dependent, with optimal performance occurring at pH 3.6. The decrease in absorbance is directly proportional to the concentration of antioxidant species present in the sample. The FRAP reagent was prepared by combining 2.5 mL of a 10 mM TPTZ solution in 40 mM HCl, 2.5 mL of a 20 mM FeCl_3_ solution, and 25 mL of 300 mM NaOAc buffer (pH 3.6). Next, 1.8 μL of the FRAP reagent was mixed with 200 μL of deionized water and 40 μL of the sample extracts. The reaction mixture was allowed to stand for 4 minutes at room temperature, after which the absorbance at 593 nm was measured. The values were determined from a calibration curve prepared using fixed concentrations of Trolox solutions, following the same procedure used for the sample extracts. The Trolox stock solution was 1,000 µM and the range of the calibration curve was 0-800 µM. Results were expressed as Trolox Equivalent (T.E.) per unit weight.

### 2.3 Statistical analysis

One-way ANOVA statistical analysis was performed with the Statistical Analysis System software using the General Linear Model (GLM Proc of the SAS Software; SAS 9.1; SAS Institute, Cary, NC, USA). The mean comparison was performed using the Tukey test (p=0.05).

## 3 Results

### 3.1 Plant bio-morphology

Plant height was the lowest in T0 plants (19.3 ± 3.4 cm) than in saline-treated plants (27.4 ± 3.1 and 25.3 ± 2.8 cm, respectively, in T100 and T400). The fresh and dry weights of shoots, stems, and leaves of *C. maritima* were significantly higher in the plants irrigated with a nutrient solution containing 100 mM NaCl (T100) than those irrigated with a solution containing 400 mM NaCl (T400). The only exception was the stem dry weight, which did not differ between salinity levels. Control plants (T0) exhibited intermediate values for these traits. Leaf number remained unchanged despite salinity levels; however, leaf area and specific leaf area (SLA) were highest in T100 and T0 plants. In contrast, leaf mass per area (LMA) and the succulence index were greatest in T400 plants, while T0 plants exhibited intermediate values. The leaf and stem dry matter (DM) were lowest in T100, whereas T400 plants had the lowest water content (**Table 1**).

**Table 1:** Growth and bio-morphological traits of shoots of *Cakile maritima* as affected by salinity.

Saline treatment ^¥^	Fresh weight (fw)			Dry weight (dw)			Leaf					Dry matter		Water content	
	Shoot	Stem	Leaf	Shoot	Stem	Leaf	Number	Area	Leaf area	Mass area	Succulence index	Leaf	Stem	Leaf	Stem
	(g)						(no.)	(cm^2^)	(cm^2^ g^-1^ dw)	(mg dw cm^-2^)	(mg fw cm^-2^)	(g kg^-1^ fw)		g 100 g^-1^	
T0	15.5 ab^1^ (± 1.3)	5.6 ab (± 0.6)	9.6 ab (± 0.9)	1.54 ab (± 0.14)	0.68 a (± 0.07)	0.86 ab (± 0.08)	37.6 a (± 2.7)	2.4 b (± 0.2)	107.3 b (± 4.9)	9.7 ab (± 0.5)	100.4 ab (± 4.1)	88.0 a (± 1.4)	123.2 ab (± 3.0)	91.2 a (± 0.02)	87.7 ab (± 0.7)
T100	18.1 a (± 1.4)	5.9 a (± 0.5)	12.0 a (± 0.8)	1.75 a (± 0.15)	0.69 a (± 0.06)	1.06 a (± 0.09)	40.8 a (± 2.9)	3.1 a (± 0.2)	125.5 a (± 6.5)	8.4 b (± 0.5)	86.9 b (± 3.3)	87.8 b (± 2.8)	115.0 b (± 4.4)	91.2 a (± 0.01)	88.5a (± 0.7)
T400	13.1 b (± 1.0)	4.3 b (± 0.3)	8.7 b (± 0.7)	1.39 b (± 0.09)	0.56 a (± 0.03)	0.83 b (± 0.07)	35.1 a (± 1.8)	2.2 b (± 0.2)	92.9 b (± 5.6)	11.7 a (± 1.0)	109.8 a (± 7.9)	95.8 a (± 1.1)	133.2 a (± 2.5)	90.4 b (± 0.01)	86.7 b (± 0.6)
Significance^2^	**	*	**	*	ns	*	ns	**	**	**	**	***	**	***	**

^1^Means (± standard error) (n=27) in columns not sharing the same letters are significantly different according to the Tukey test (α=0.05).

^2^Significance: *, **, *** for P ≤ 0.05, ≤ 0.01 and ≤ 0.001, respectively; ns, not significant.

^¥^T0 = 0 mM NaCl; T100 = 100 mM NaCl; T400 = 400 mM NaCl.

The root apparatus of plants treated with 100 mM NaCl exhibited greater fresh weight, length, and volume, especially when compared to T400 plants. However, T100 plants had the lowest root DM and higher water content (WC). Root dry weight was similar across treatments, and no differences were observed in root diameter or shoot:root dry weight ratio (**Table 2**).

**Table 2:** Growth and bio-morphological traits of root apparatus of *Cakile maritima* as affected by salinity.

Saline treatment^¥^	Root fresh weight	Dry matter	Dry weight	Root length	Root volume	Root diameter	Shoot: Root	Water content
	(g)	(g kg^-1^ fw)	(g)	(cm)	(cm^-3^)	(mm)	(g dw g^-1^dw)	g 100 g^-1^
T0	0.83 ab^1^ (± 0.8)	153.8 b (± 0.2)	0.12 a (± 0.02)	632.5 b (± 121.0)	0.8 ab (± 0.2)	0.41 a (± 0.04)	16.3 a (± 1.9)	84.6 b (± 0.3)
T100	0.87 a (± 0.11)	126.9 c (± 0.3)	0.11 a (± 0.02)	1012.3 a (± 108.2)	1.1 a (± 0.2)	0.37 a (± 0.02)	26.2 a (± 6.2)	87.2 a (± 0.3)
T400	0.56 b (± 0.07)	191.5 a (± 0.4)	0.10 a (± 0.01)	641.9 b (± 87.8)	0.6 b (± 0.1)	0.34 a (± 0.02)	19.7 a (± 6.3)	80.5 c (± 0.5)
Significance^2^	*	**	ns	**	*	ns	ns	***

^1^Means (± standard error) (n=27) in columns not sharing the same letters are significantly different according to the Tukey test (α=0.05).

^2^Significance: *, **, *** for P ≤ 0.05, ≤ 0.01 and ≤ 0.001, respectively; ns, not significant.

^¥^T0 = 0 mM NaCl; T100 = 100 mM NaCl; T400 = 400 mM NaCl.

### 3.2 Mineral concentrations

Leaf chloride, nitrate, potassium, calcium, and magnesium concentrations were not affected by the treatments. In contrast, sodium concentration was higher in the leaves of saline-treated plants, regardless of NaCl level (**Table 3**). In the stems, the T400 plants exhibited the highest sodium concentration, while the T0 plants showed the greatest calcium level (**Table 4**).

**Table 3:** Inorganic ion concentrations in leaves of *Cakile maritima* as affected by salinity.

Saline treatment^¥^	Cl	N0_3_	Na	K	Mg	Ca
	mg kg^-1^ dw					
T0	1233 b^1^ (± 254)	5401 a (± 1124)	8758 b (± 649)	19396 a (± 1586)	3352 a (± 269)	26054 a (± 2438)
T100	2493 a (± 405)	5441 a (± 937)	16133 a (± 807)	20577 a (± 1028)	4235 a (± 336)	25975 a (± 1884)
T400	2156 a (± 256)	4538 a (± 773)	15782 a (± 1344)	20437 a (± 1100)	3360 a (± 262)	24959 a (± 2040)
Significance^2^	**	ns	***	ns	ns	ns

^1^Means (± standard error) (n=27) in columns not sharing the same letters are significantly different according to the Tukey test (α=0.05).

^2^Significance: **, *** for P ≤ 0.01 and ≤ 0.001, respectively; ns, not significant.

^¥^T0 = 0 mM NaCl; T100 = 100 mM NaCl; T400 = 400 mM NaCl.

**Table 4:** Cation concentrations in stems of *Cakile maritima* as affected by salinity.

Saline treatment¥	Na	K	Mg	Ca
	mg g^-1^ dw			
T0	36.1 b^1^ (± 3.0)	35.8 a (± 3.1)	41.0 a (± 3.7)	186.6 a (± 10.1)
T100	36.8 b (± 3.3)	35.3 a (± 1.7)	29.0 a (± 5.7)	111.5 b (± 7.6)
T400	67.4 a (± 3.9)	32.1 a (± 2.5)	34.6 a (± 4.9)	132.6 b (± 6.9)
Significance^2^	***	ns	ns	***

^1^Means (± standard error) (n=27) in columns not sharing the same letters are significantly different according to the Tukey test (α=0.05).

^2^Significance: *** for p ≤ 0.001; ns, not significant.

^¥^T0 = 0 mM NaCl; T100 = 100 mM NaCl; T400 = 400 mM NaCl.

### 3.3 Photosynthetic gas exchange and chlorophyll fluorescence

Stomatal conductance (g_s_) and transpiration (E) were unaffected by salinity. However, intercellular CO_2_ concentration (C_i_) decreased in T100 and T400 leaves, coinciding with a higher net CO_2_ assimilation rate (A_n_), particularly in T100. The operating efficiency of PSII in the light (ΦPSII) did not differ between treatments, but the maximum PSII photochemical efficiency in the light (Fv’/Fm’) was highest in T0 leaves. Photochemical quenching (qP) was lower in T0, especially compared to T400 (**Table 5**).

**Table 5:** Photosynthetic gas exchange and chlorophyll fluorescence of leaf of *Cakile maritima* as affected by salinity.

Saline treatment^¥^	Net assimilation rate (A_n_)	Stomatal conductance to H_2_O (g_s_)	Intercellular CO_2_ concentration (C_i_)	Transpiration (E)	Photochemical Quencing (qP)	Maximum PSII efficiency (Fv’/Fm’)	Operating PSII efficiency (ΦPSII)
	(μmol CO_2_ m^-2^ s^-1^)	(mol H_2_O m^-2^ s^-1^)	(μmol CO_2_ mol air^-1^)	(mmol H_2_O m^-2^ s^-1^)	(-)	(-)	(-)
T0	3.64 b^1^ (± 0.04)	0.06 a (± 0.01)	282.5 a (± 18.2)	1.9 a (± 0.2)	0.19 b (± 0.04)	0.50 a (± 0.02)	0.10 a (± 0.02)
T100	6.04 a (± 1.24)	0.07 a (± 0.01)	243.4 b (± 7.6)	2.1 a (± 0.2)	0.25 ab (± 0.02)	0.42 b (± 0.02)	0.11 a (± 0.01)
T400	4.57 ab (± 0.86)	0.05 a (± 0.01)	247.3 b (± 13.6)	1.6 a (± 0.3)	0.30 a (± 0.04)	0.43 b (± 0.02)	0.13 a (± 0.01)
Significance^2^	*	ns	*	ns	*	*	ns

^1^Means (± standard error) (n=18) in columns not sharing the same letters are significantly different according to the Tukey test (α=0.05).

^2^Significance: * for p ≤ 0.05; ns, not significant.

^¥^T0 = 0 mM NaCl; T100 = 100 mM NaCl; T400 = 400 mM NaCl.

### 3.4 Pigment, bioactive compound concentrations and antioxidant capacity

Chlorophyll *a* level decreased in T100 and T400 leaves, which also impacted the total concentration of these pigments. In contrast, chl *b* and carotenoid concentrations remained unchanged. Ascorbic acid, flavonoid, and anthocyanin levels were highest in T400 leaves, with T100 showing a significant decline in ascorbic acid (**Table 6**). Total phenols (TP) and antioxidant capacity (DPPH, ABTS, FRAP assays) were generally unaffected by treatments, except for lower TP and ABTS values in T400 leaves. Since lipophilic components were negligible, total antioxidant capacity was calculated as the sum of hydrophilic and lipophilic fractions (**Table 7**).

**Table 6:** Pigment and bioactive compound concentrations in leaves of *Cakile maritima* as affected by salinity.

Saline treatment^¥^	Chlorophyll			Carotenoids	Ascorbic acid	Phenols	Flavonoids	Anthocyanins
	*a*	*b*	Total	(mg 100 g^-1^ dw)		(mg a.g.e 100 g^-1^ dw)^3^	(mg q.e. 100 g^-1^ dw)^3^	(mg c.g. 100 g^-1^ dw)^3^
	(µg g^-1^ dw)							
T0	165.0 a^1^ (± 9.6)	84.5 a (± 6.3)	241.8 a (± 14.9)	0.24 a (± 0.01)	4.4 b (± 0.3)	656.3 a (± 26.1)	2.1 b (± 0.1)	72.3 b (± 5.4)
T100	137.7 b (± 9.5)	84.6 a (± 4.1)	213.4 b (± 11.8)	0.25 a (± 0.01)	3.8 c (± 0.1)	656.6 a (± 42.5)	2.2 b (± 0.04)	76.0 b (± 3.5)
T400	135.1 b (± 7.4)	90.4 a (± 6.7)	215.6 b (± 12.2)	0.27 a (± 0.01)	5.2 a (± 0.4)	482.9 b (± 20.7)	2.7 a (± 0.1)	88.0 a (± 5.5)
Significance^2^	***	ns	**	ns	***	***	***	**

^1^Means (± standard error) (n=18) in columns not sharing the same letters are significantly different according to the Tukey test (α=0.05).

^2^Significance: *** and **, respectively, for p ≤ 0.001 and, p ≤ 0.01; ns, not significant.

^3^a.g.e=acid gallic equivalent; q.e.=quercetin equivalent; c.g.=cyanidin-3-glucoside equivalent.

The meaning of the symbol ¥ is T0= 0 mM NaCl; T100=100 mM NaCl; T400= 400 mM NaCl.

**Table 7:** Leaf antioxidant capacity of *Cakile maritima* as affected by salinity.

Saline treatment^¥^	Antioxidant capacity		
	DPPH	ABTS	FRAP
	(µmol T.E^3^. g^-1^ dw)		
T0	13.8 a^1^ (± 0.4)	46.3 a (± 1.7)	51.7 a (± 1.4)
T100	14.2 a (± 1.1)	47.5 a (± 2.1)	53.4 a (± 1.9)
T400	13.7 a (± 0.7)	35.9 b (± 1.5)	56.5 a (± 3.4)
Significance^2^	ns	***	ns

^1^Means (± standard error) (n=18) in columns not sharing the same letters are significantly different according to the Tukey test (α=0.05).

^2^Significance: *** for p ≤ 0.001; ns, not significant.

^3^T.E.=trolox equivalent.

¥ T0= 0 mM NaCl; T100=100 mM NaCl; T400= 400 mM NaCl.

## 4 Discussion

### 4.1 Growth and morpho-nutritional responses

In this study, the best growth was observed for T100 plants. The aerial part produced the highest fresh (+40%) and dry (+38%) biomass. The latter, primarily allocated to the leaves. The increase in leaf biomass was due to expansion rather than an increase in the number of leaves (**Table 1**). Plants grown at 100 mM NaCl also produced a root apparatus more developed with longer roots, but with lower dry matter (DM), suggesting a trade-off between growth and resource allocation. Although the shoot:root ratio trended higher in T100, the difference was not significant (**Table 2**).

Compared to control plants (T0), the most notable differences, rather than shoot and root dry and fresh biomass production, involved shoot height, leaf expansion and root length, ultimately leading to dry mass dilution in these organs. The shoot water content remained unchanged (leaves) or slightly increased (stems), and it even improved in roots (**Tables 1**, **2**).

This research represents the first investigation into the saline responses of a *C. maritima* (pinnatifid) ecotype from Italy. To date, previous studies have focused solely on Tunisian *C. maritima* populations, reporting findings that are partially consistent with ours. When evaluating multiple NaCl concentrations, many authors (Debez et al., 2004, 2006, 2008, 2012; Yepes et al., 2018; Ben Amor et al., 2020) reported an improvement of dry biomass for plants nourished with 100 mM NaCl. However, others observed slight or no changes in plant growth (Ben Amor et al., 2006, 2007; Ben Hamed-Louati et al., 2016; Arbelet-Bonnin et al., 2020), with responses varying depending on the ecotype (Ben Amor et al., 2006; Ksouri et al., 2007; Megdiche et al., 2007). Moreover, these studies often found an enhancement of leaf number (Debez et al., 2004, 2008, 2012) rather than leaf area (Ksouri et al., 2007; Megdiche et al., 2007) passing from 0 to 100 mM NaCl.

Accounting for other morphological traits, the leaves of T100 plants tended to be thinner (exhibiting higher SLA and lower LMA) and less succulent than T0, despite having similar water content (**Table 1**). These bio-morphological traits were not strictly in line with previous research, which reported an improvement of plant water status along with succulence (Debez et al., 2004, 2006, 2012) and leaf thickness (Debez et al., 2006; Yepes et al., 2018). These changes occurred in response to increasing Na (and Cl) uptake and accumulation in leaves observed starting from a 50–100 mM NaCl concentration in the substrate.

Succulence mechanisms are the main strategy that salt-including halophytes (euhalophytes) adopt under saline conditions to maintain cell turgor pressure and prevent salt accumulation in the cytosol. It includes several biochemical responses such as ion homeostasis and osmotic adjustments, both involving Na, Cl and K regulation in the plant. The succulence of plants arises from solute and water storage in vacuoles, along with the corresponding osmolyte concentrations in the cytosol (Debez et al., 2012, 2013; Farhat et al., 2021; Ellouzi et al., 2011, 2014; Mir et al., 2024). Ultimately, this adaptive strategy results in tissues with enhanced water content, characterized by thick leaves and stems that exhibit large mesophyll cells and large vacuoles, ensuring Na and Cl dilution and maintaining photosynthetic efficiency (Rahman et al., 2021).

In *C. maritima* grown at up to 400 mM NaCl, the compartmentalization of excess sodium into vacuoles has been demonstrated to maintain cytosolic Na homeostasis (Debez et al., 2004, 2006; Ben Hamed-Louati et al., 2016). It was linked to the activity of vacuolar H+-ATPase, which creates a pH gradient between the cytoplasm and the vacuole, allowing Na transport against the electrochemical gradient (Debez et al., 2004), thus limiting the increase of Na/K ratio in the cytosol.

In the case of the Italian *C. maritima* ecotype, the accumulation of Na and Cl in leaves at T100 and the contemporary plant growth and balanced water status prove that these ions were excluded from the cytosol and likely were stored in the vacuoles, confirming the findings of other Authors (Debez et al., 2004, 2006; Ben Hamed-Louati et al., 2016). The different responses in terms of leaf thickness and succulence (**Table 1**) could be linked to the level of salt accumulation. In our research, the Na and Cl leaf concentrations increased by 84% and 102%, respectively, in comparison to T0 (**Table 3**); nevertheless, both Na (0.7 mmol g^-1^ DW) and Cl (0.03 mmol g^-1^ DW) were markedly lower than those reported for Tunisian populations (Debez et al., 2004, 2008, 2012; Ellouzi et al., 2011; Ben Hamed-Louati et al., 2016; Ben Amor et al., 2020) grown at the same saline treatment. Leaf Na and Cl concentrations similar to ours were reported only by Houmani et al. (2023), despite these authors not indicating ecotype provenience. Overall, these findings indicate that there are specific thresholds for inducing succulence that vary by ecotype.

Specifically, the reduction of leaf succulence was due to its expansion, which, along with root elongation, mainly contributed to the growth improvement in T100 plants (**Tables 1**, **2**). Supporting this finding, a proteomic analysis of the Tunisian genotype of *C. maritima*, exposed to a salinity level of 100 mM NaCl, revealed an abundance of proteins potentially linked to cell growth (putative elongation chloroplast factor). The increase in the expression of this protein was associated with the greater leaf initiation and expansion compared to plants grown at 0 mM NaCl (Debez et al., 2012).

Differences in growth were significant when comparing T100 and T400 plants, with the latter not exhibiting visible damage. The T400 plants, despite having similar height, showed a reduction in shoot fresh (-28%) and dry (-21%) biomass, root fresh biomass (-36%) and length, as well as in leaf, stem, and root water content. In T400, leaves were thicker (higher LMA) and more succulent, exhibiting all typical morphological adaptations of halophytes to soil salinity (**Tables 1**, **2**). Roots maintained stable dry biomass, suggesting that salts were not accumulated in the roots but could have been exported in the aerial parts, confirming the behavior observed in other ecotypes of *C. maritima* (Ben Hamed-Louati et al., 2016). It is noteworthy that the growth inhibition effect of 400 mM NaCl on *C. maritima* ecotype under study was milder than in other ecotypes (Debez et al., 2004, 2008; Ben Amor et al., 2006; Ksouri et al., 2007; Ben Hamed-Louati et al., 2016; Yepes et al., 2018; Arbelet-Bonnin et al., 2020).

No changes were detected in Na and Cl concentrations in T100 and T400 leaves (**Table 3**). If a Cl saturation at 50 mM NaCl level has been observed by Debez et al. (2004), the lack of increase in leaf sodium concentration with rising substrate salinity was unexpected, even though the T400 leaves typically exhibited succulence. Conversely, our results point out that Na accumulation occurred in the stems (green + mature), where it was enhanced by 83% in the T400 treatment. This suggests that stems serve as a primary Na sink in this ecotype (46.8 vs 13.5 mg g^-1^ DW, on average) (**Table 4**), diverging from prior studies that emphasize leaf accumulation (Debez et al., 2008).

Further investigation is needed to explore the overall low sodium content in the shoots of this ecotype of *C. maritima* and its partitioning in the different parts of the plant. Studies conducted on Italian morphotypes of *C. maritima* have indicated that salt-excreting structures are present in pinnatifid-leaf types (Ciccarelli et al., 2010). Previous research on the same pinnatifid morphotype of this study revealed a lower Na concentration in the shoots compared to the entire-leaf type (Conversa et al., 2024). Therefore, a more in-depth evaluation of leaf morphology is necessary to determine the presence of salt-excreting structures and their potential role in reducing Na content in the leaves. Additionally, along with the possible contribution of Na excretion, in future research, it is important to consider a Na-excluding mechanism at the root level. To deal with salt stress, the most significant signaling pathway is the salt overly sensitive (SOS), which requires the combined activities of three proteins, namely SOS3, SOS2, and SOS1 (a plasma membrane-localized Na+/H+ antiporter) expelling Na out of the cell (Shabala and Mackay, 2011; Ji et al., 2013; Shabala et al., 2015; Julkowska and Testerink, 2015). In *C. maritima* culture cells, the constitutive expression of the SOS has been determined, which could be involved at the root level not only in Na upload in the xylem leading to shoot accumulation, but also in salt exclusion from cells (Arbelet-Bonnin et al., 2018).

The stable concentration, from 0 up to 400 mM NaCl, of K and Mg in leaves and stems means that no impairment in uptake of these cations occurred at both saline levels. These findings seem to confirm the selective K uptake pointed out in Tunisian *C. maritima* ecotypes, counteracting the Na excess (Debez et al., 2004, 2006, 2008; Ben Hamed-Louati et al., 2016). Despite these authors (except Ben Hamed-Louati et al., 2016) reporting a reduction of K, Ca and Mg concentration, they never observed nutritional disruption in salt-exposed plants. The regulation of the uptake and transport of Na and K (and Mg) in the plant is essential in halophytes when facing salinity. This regulation helps maintain an optimal K/Na ratio in the cytosol since potassium is involved in various metabolic processes and osmotic adjustment. Specifically, while Na replaces K in the vacuoles to serve as an osmotic agent, potassium, together with compatible organic solutes, plays a vital role in the cytosol to maintain cell turgor pressure (Rahman et al., 2021).

Calcium was the most abundant cation, especially in the stems (see **Tables 3**, **4**). A higher concentration of calcium compared to sodium in the leaves was noted by Yepes et al. (2018), although this finding contradicts other studies (Debez et al., 2004; Ellouzi et al., 2011). It remained unchanged in the leaves but showed a reduction in stems of salt-treated plants, irrespective of NaCl level. Exposure to NaCl triggers an increase in cytosolic Ca concentration, which is essential for activating plasma membrane H+-ATPases, ultimately leading to the stimulation of the SOS pathway (Ji et al., 2013; Shabala et al., 2015). Such a phenomenon has been observed in the cell suspension of *C. maritima* (Arbelet-Bonnin et al., 2018). Consequently, the decrease in calcium levels in accumulation sites (stems) when plants are exposed to salinity could indicate that leaves may act as a sink for this cation, playing a role in sodium regulation via the SOS pathway. On the other hand, a moderate calcium level has been proven to enhance the tolerance of *C. maritima* under salt stress by increasing several antioxidative enzyme activities (Ben Amor et al., 2010).

### 4.2 Photosynthetic responses

The plant growth in saline-tolerant genotypes, along with effective Na, Cl, and K regulation, is strictly dependent on efficient gas exchange and scavenging of reactive oxygen species (ROS) to ultimately preserve photosynthesis apparatus efficiency for producing plant dry biomass.

Salt stress typically impairs photosynthesis by both stomatal (e.g. CO_2_ uptake) and non-stomatal (e.g. chlorophyll, Rubisco) limitation, leading to excessive energy exposure of plants beyond their capacity to utilize it in photosynthesis. Therefore, one of the physiological adaptive mechanisms to salinity is the protection of the photosynthetic apparatus through the dissipation of energy excess and ROS detoxification (Qiu et al., 2003; Debez et al., 2008; Megdiche et al., 2008; Mann et al., 2023).

In the current study, the ΦPSII parameter (operating efficiency of PSII in the light), which indicates the fraction of the absorbed light actually used for photochemistry processes (Murchie and Lawson, 2013), remained stable across treatments. However, its components Fv’/Fm’ and qP parameters highlighted photosynthetic adjustments in plants treated with NaCl (**Table 5**). The maximum PSII photochemical efficiency in the light (Fv’/Fm’), representing the efficiency if all PSII centers were open, was the lowest in both saline-treated plants. Since any decrease of Fv’/Fm’ is inversely related to the dissipation of energy excess mainly as heat (non-photochemical quenching -NPQ) (Baker, 2008; Maxwell and Johnson, 2000), it can be argued that salt-treated plants exhibited higher NPQ as a photosynthetic apparatus protective strategy (Malnoë, 2018).

A reduction of Fv’/Fm’ was observed in *C. maritima* at 400 mM NaCl (Arbelet-Bonnin et al., 2020), while higher NPQ levels were also revealed in plants under saline conditions starting from 100 to 500 mM NaCl (Debez et al., 2008; Megdiche et al., 2008; Farhat et al., 2021). Photoprotection mechanisms acting under stressful conditions, such as salinity, involve various processes at the chloroplast level, including carotenoid and anthocyanin pigments (Megdiche et al., 2008). Carotenoids absorb short-wavelength light and transfer energy to chlorophylls, preventing the formation of chlorophyll triplets and reactive oxygen species (ROS). Anthocyanins effectively filter visible light, thus preventing the production of ROS that can be induced by excessive light (Ashikhmin et al., 2023).

In our study, carotenoids were not affected by salinity; however, the Car/Chl ratio improved in salt-treated plants due to chlorophyll reduction (**Table 6**). The higher Car/Chl was in line with results reported for a more salt-tolerant *C. maritima* Tunisian ecotype (Megdiche et al., 2008), confirming the role of carotenoids in avoiding the oxidative stress and photosynthesis impairment deriving from salt exposure. Reduction in chlophyll pigments was found due to a decrease in the Chl a (**Table 6**), which is reported to be more salt-sensitive than Chl b (Megdiche et al., 2008); however, it occurred only at moderate salinity levels. The rise in anthocyanins in T400 plants (**Table 6**) can be explained as an additional protective mechanism for chlorophyll and PSII. The enhancement of leaf anthocyanins in *C. maritima* under the highest salinity conditions has also been observed in many studies (Debez et al., 2008, 2012; Mansour et al., 2018; Farhat et al., 2021) as PSII protection related to a strong antioxidative response.

Despite Fv’/Fm’ reduction, the T100 and especially T400 plants showed a higher PSII efficiency factor (qP), which indicates the actual fraction of Fv’/Fm’ that is achieved. In other words, this factor is related to the proportion of open reaction centers, highlighting the level of photochemical quenching occurring in PSII. These findings suggest the capability of salt-exposed plants to maintain the efficiency of photosynthetic machinery, improving the ability to utilize light. In line with this study, unchanged qP has been reported in *C. maritima* from 0 to 400 mM NaCl (Megdiche et al., 2008). Additionally, improved qP was observed for plants grown at 200 mM NaCl compared with the control (Farhat et al., 2021).

In disagreement with our results, a reduction in ΦPSII was observed for *C. maritima* grown at salinity levels exceeding 200 mM NaCl (Debez et al., 2008) or 400 mM NaCl (Arbelet-Bonnin et al., 2020).

Whereas, comparing two ecotypes, ΦPSII decreased only in the less tolerant one at 400 mM NaCl (Megdiche et al., 2008). The ΦPSII impairment may be related to CO_2_ limitation due to stomatal closure as an adaptive response to salinity; CO_2_ limitation reduces the rate of ATP and NADPH consumption, and in turn, ΦPSII (Baker and Rosenqvist, 2004; Baker, 2008). In the case of the studied *C. maritima* ecotype, the steady ΦPSII could also be linked to unchanged stomatal conductance up to 400 mM NaCl (gs, E, **Table 5**).

The ΦPSII is linked to the linear electron flow through the reaction centers, which plays a role in CO_2_ assimilation (A_n_). However, there is often a discrepancy between the efficiency of PSII and the rate of CO_2_ fixation. This gap is primarily attributed to the phenomenon of photorespiration which is essential for protecting PSII and sustaining photosynthesis (Maxwell and Johnson, 2000; Shi et al., 2022). Indeed, photorespiration can act as a sink for photosynthetically generated electrons, especially under stress conditions (Sunil et al., 2019; Voss et al., 2013).

In this study, after nearly one month of saline stress, a significant discrepancy in A_n_ and ΦPSII was detected only for the T0 plants (**Table 5**). Since no restriction on CO_2_ can be supposed to be caused by stomata closure, in control plants, the higher C_i_ suggests lower CO_2_ consumption and the occurrence of photorespiration as an electron sink, explaining their lower net CO_2_ assimilation (-40%) versus T100 (**Table 5**). This finding can help to explain the slight reduction in growth (shoot dry biomass) of the salt-free plants (**Table 1**), highlighting that they experienced stress more than moderate salt-exposed plants. Therefore, this investigation confirms that this species is an euhalophyte, revealing the role of Na as an essential nutrient.

Interestingly, the T400 plants showed a negligible decrease in A_n_ while exhibiting gas exchange (g_s_) and CO_2_ intercellular concentration (C_i_) very similar to T100 (**Table 5**). Conversely, many Authors have highlighted that a drop in A_n_ generally occurs in *C. maritima* at salinity levels higher than 200 mM NaCl because of stomata closure to reduce water loss through transpiration (Debez et al., 2004, 2008; Megdiche et al., 2008; Ben Hamed-Louati et al., 2016; Arbelet-Bonnin et al., 2020). It is surprising to find unchanged stomatal conductance and transpiration (E) (**Table 5**). This phenomenon, observed at 100 mM NaCl, has been related to the replacement of K with Na in guard cells, which helps maintain stable turgor pressure (Debez et al., 2006). In our study, this mechanism could have occurred effectively even at salinity levels exceeding 100 mM NaCl, thereby preventing any restriction on CO_2_ uptake. Moreover, leaf anatomical features of a pinnatifid ecotype (large and outwardly turned epidermal vesicular cells, cell guards rich in essential oils) (Ciccarelli et al., 2010) have been reported to assure abiotic stress tolerance under natural habitat by sustaining higher transpiration rates (Conversa et al., 2024). They could also be involved in the salt responses. However, this aspect warrants further research to confirm and elucidate the unexpected salt-adaptive mechanisms of this *C. maritima* ecotype.

Although photosynthesis per unit leaf does not seem affected by higher salinity, the reduced leaf area of T400 plants leads to diminished photosynthesis at the canopy level, resulting in decreased shoot growth and dry biomass production. Likely this occurred for sustaining energy-requiring ion compartmentalization, osmoregulation, morphological adaptation, and antioxidative responses to salinity (Debez et al., 2006; Rahman et al., 2021; Bazihizina et al., 2024) observed in these plants. Additionally, an osmotic effect can be argued due to the enhancement of dry mass concentration and reduction in water content in leaves, stems and roots (**Tables 1**, **2**).

### 4.3 Non-enzymatic antioxidative leaf status

Concerning antioxidant compounds, both carotenoids and anthocyanins may act as antioxidants directly scavenging ROS, along with other compounds such as ascorbate, phenols, flavonoids and glutathione (Rahman et al., 2021; Mann et al., 2023). Specifically, we found under supra-optimal salinity conditions an increase of flavonoids, anthocyanins and ascorbate (AsA) (**Table 6**). These results are consistent with prior reports for anthocyanins in this species. Contrarily, ascorbate reduction at increasing salinity is reported by many Authors (Ben Amor et al., 2006, 2007; Ben Hamed-Louati et al., 2016; Mansour et al., 2018) despite others having found that it was improved at 400 mM NaCl at the chloroplast level (Ben Amor et al., 2020). On the other hand, a higher AsA retention passing from 0 to 400 mM NaCl was observed in a more salt-tolerant *C. maritima* genotype (Ben Amor et al., 2006).

Ascorbate is the main biologically active form of vitamin C, as it interacts with ROS, undergoing oxidation to dehydroascorbic acid (DHAA); in plants, DHAA is either recycled to AsA by the dehydro-ascorbate reductase, using glutathione as the reductant, or irreversibly hydrolyzed. Efficient ascorbate recycling (high AsA/DHAA) may be critical to maintain ROS at a level that minimizes oxidative damage (Gallie, 2013). Although we did not detect DHAA for evaluating the changes of AsA/DHAA in salt-free and salt-treated plants, the highest AsA in T400 may suggest an improved antioxidative defense of these plants. An increase in AsA was found in halophytes *C. maritima* and *Thellungiella salsuginea* within 72 h to 400 mM NaCl. It was related to an earlier response to stress imposition compared to a glycophyte (*Arabidopsis thaliana*), with an efficient DHAA recycling (Ellouzi et al., 2014).

Among other antioxidant compounds, an increase in TP content in salt-exposed plants was expected, as it is involved in counteracting ROS resulting from abiotic stresses (Conversa et al., 2021). In our study, TP diminished at 400 mM NaCl with no changes between control and mild-saline treatment (**Table 6**). Partially in line with this study, in the more salt-tolerant sea rocket ecotype, TP accumulation drops from 100 to 400 mM NaCl, despite an enhancement occurring from 0 to 100 mM NaCl; in the less salt-tolerant sea rocket ecotype, TP accumulation was lower and unaffected by salinity (Ksouri et al., 2007). On the contrary, TP accumulation progressively increased under salinity constraints in another *C. maritima* Tunisian ecotype due to the activity of the key enzyme for their biosynthesis (Mansour et al., 2018).

In the euhalophytes *Salicornia* and *Sarcocornia* species, it has been demonstrated that, after an increase in TP at moderate salt levels (35–200 mM NaCl), plants lose the ability to produce these compounds, activating other protective mechanisms such as osmolyte synthesis and/or salt exclusion (Lima et al., 2020). Considering the morphological and physiological adaptation described above for the T400 plants, a shift of their resources towards other metabolic mechanisms for preventing ROS production or providing their detoxification could be plausible. Antioxidant capacity (AC) assays (FRAP, DPPH, ABTS) showed no salinity-linked trends (**Table 7**). Only ABTS revealed a relationship with TP changes, as expected (Conversa et al., 2019). There is limited and non-univocal published data regarding the link between antioxidant compounds and antioxidant capacity under saline treatments (Ksouri et al., 2007; Mansour et al., 2018). As a result, specific antioxidants and AC tests do not seem to fully capture the plant’s response to salinity. To obtain a comprehensive analysis, the enzymatic antioxidative system should be explored, as it is a strong indicator of oxidative stress (Ben Amor et al., 2006, 2007; Arbelet-Bonnin et al., 2019). Additionally, further research into bioactive compounds is needed, as their potential benefits to humans are significant. Extracts from whole wild *C. maritima* plants demonstrated high DPPH radical scavenging activity due to several bioactive compounds, which were positively correlated with their anti-proliferative effects on CaCo2 and HeLa carcinoma cell lines (Omer et al., 2019). The DPPH of the leaves and stems of *C. maritima* was associated with total phenolic content and exhibited anti-inflammatory, anti-bacterial, and anti-proliferative activities against multiple myeloma cells (Fuochi et al., 2019). These extracts showed inhibitory properties against key enzymes involved in human diseases (Placines et al., 2020).

## 5 Conclusions

Our investigation of the Apulian ecotype of C. maritima confirms its classification as a sodium-including halophyte, demonstrating optimal growth at moderate salinity levels of 100 mM NaCl. Under these conditions, we observed a significant sodium accumulation in leaves, exceeding 80% compared to non-saline controls. The leaf Na level optimal for growth remained consistent even at higher salt concentrations (400 mM NaCl), as the excess sodium was accumulated in the stems, proving the ability is this ecotype to maintain leaf sodium concentrations conducive to growth even at higher salt concentrations. At 400 mM NaCl, this adaptation allows plant growth, albeit at slightly reduced rates, with the plants showing characteristic morpho-physiological adjustments like increased leaf succulence while displaying no visible toxicity symptoms. The study reveals another noteworthy aspect regarding essential cations, with K, Mg, and Ca uptake levels remaining largely stable, indicating the presence of an efficient ion uptake regulation system that prevents nutritional imbalances under high salinity stress.

Although this represents an initial characterization of this specific ecotype, our findings clearly show these plants avoid severe growth limitations by maintaining effective stomatal regulation and preserving photosynthetic apparatus functionality under saline stress. To fully understand this ecotype’s potential, future research should explore the physiological mechanisms enabling water balance maintenance and sustained photosynthetic energy production, particularly during prolonged salinity exposure. Additional investigations could clarify the molecular basis of these adaptations and evaluate potential applications in marginal agriculture or soil salinization scenarios.

## Data Availability

The raw data supporting the conclusions of this article will be made available by the authors, without undue reservation.
