# Supplementary material for: Salinity tolerance in the halophyte species Cakile maritima from the Apulia region, southern Italy
Source: Front Plant Sci. 2025 Sep 1;16:1662491. doi: 10.3389/fpls.2025.1662491 (PMC12438835; doi:10.3389/fpls.2025.1662491)
Supplement: Supplementary file 1 [file Table1.docx]

Supplementary Table 1 – Physicochemical properties of sandy soil from the dunes of Margherita di Savoia (BT, Italy), a natural site where *Cakile maritima* can be commonly found.

| **Determination** | **Unit** | **Result** |
| --- | --- | --- |
| Bulk density | (kg m^-3^) | 1470 |
| Silt | (%) | 0 |
| Sand | (%) | 93.8 |
| Clay | (%) | 6.2 |
| pH |  | 7.9 |
| EC | µS cm^-1^ | 950 |
| Cation exchange capacity | (mmoli kg^-1^) | 24 |
| Organic matter | (g 100 g^-1^) | 0.5 |
| Total nitrogen | (g kg^-1^) | 0.48 |
| Exchangeable potassium (K_2_O) | (mg kg^-1^) | 50.6 |
| Assimilable phosphorus (P_2_O_5_) | (mg kg^-1^) | 8.5 |
| Available calcium (Ca) | (mg kg^-1^) | 79 |
| Available magnesium (Mg) | (mg kg^-1^) | 43 |
| Available sodium (Na) | (mg kg^-1^) | 48 |
| Chloride (Cl) | (mg kg^-1^) | 99.3 |
| Available sulfur (S) | (mg kg^-1^) | 21.2 |
| Assimilable iron (Fe) | (µg kg^-1^) | < 2010 |
| Available silicon (Si) | (µg kg^-1^) | 24030 |
| Assimilable manganese (Mn) | (µg kg^-1^) | 470 |
| Assimilable zinc (Zn) | (µg kg^-1^) | < 100 |
| Assimilable copper (Cu) | (µg kg^-1^) | 33 |
| Soluble boron (B) | (µg kg^-1^) | 212 |
| Available cobalt (Co) | (µg kg^-1^) | < 2.6 |
| Available molybdenum (Mo) | (µg kg^-1^) | 3 |
