# Supplementary material for: Salinity tolerance in the halophyte species Cakile maritima from the Apulia region, southern Italy
Source: Front Plant Sci. 2025 Sep 1;16:1662491. doi: 10.3389/fpls.2025.1662491 (PMC12438835; doi:10.3389/fpls.2025.1662491)
Supplement: Supplementary file 2 [file Table2.docx]

Supplementary Table 2 – Chemical characteristics of tap water used for *C. maritima* plants irrigation and to produce T0, T100 and T400 nutrient solutions.

| Parameter | Unit of measurement | Value | Law limit^*^ |
| --- | --- | --- | --- |
| Electric Conductivity (EC) | µS cm^-1^ at 20°C | 582 | 2500 |
| pH |  | 7.9 | 6.5 ≤ x ≤ 9.5 |
| Nitrate (NO₃) | mg L^-1^ | 3 | 50 |
| Potassium (K) | mg L^-1^ | 7 | - |
| Calcium (Ca) | mg L^-1^ | 50 | - |
| Magnesium (Mg) | mg L^-1^ | 16 | - |
| Sodium (Na) | mg L^-1^ | 58 | 200 |
| Chloride (Cl) | mg L^-1^ | 44 | 250 |
| Boron (B) | mg L^-1^ | 0.1 | 1.5 |

^*^ Legislative Decree 18/2023
